# SRC-3 Knockout Attenuates Myocardial Injury Induced by Chronic Intermittent Hypoxia in Mice

**DOI:** 10.1155/2021/6372430

**Published:** 2021-11-03

**Authors:** Wanyu Wang, Hongbo Gu, Weihua Li, Yihua Lin, Xiangyang Yao, Wen Luo, Fang Lu, Shenhui Huang, Yonghong Shi, Zhengrong Huang

**Affiliations:** ^1^Department of Pulmonary and Critical Care Medicine of the First Affiliated Hospital of XiaMen University, The Third Clinical Medical College of Fujian Medical University, Teaching Hospital of Fujian Medical University, Xiamen, China; ^2^Department of Cardiology, Affiliated Hospital of Weifang Medical University, Weifang, China; ^3^Department of Cardiology, Xiamen Key Laboratory of Cardiac Electrophysiology, Xiamen Institute of Cardiovascular Diseases, the First Affiliated Hospital of Xiamen University, School of Medicine, Xiamen University, Xiamen, China; ^4^Department of Pulmonary Diseases of Xinglin branch of the First Affiliated Hospital of Xiamen University, Xiamen City, China

## Abstract

This study investigated the effects of chronic intermittent hypoxia (CIH), a model of sleep apnea syndrome (SAS), on cardiac function. SRC-3 was extremely lowly expressed in the adult mouse heart tissue, while SRC-3 was highly expressed in the adult mouse heart tissue after CIH, suggesting that SRC-3 is involved in CIH model. We further studied the role of SRC-3 in CIH-induced myocardial injury in mice. Twenty-four healthy Balb/c male mice (*n* = 16, wild type; *n* = 8, SRC-3 knockout (SRC3-KO)) were randomly divided into three groups: air control (Ctrl), CIH, and CIH+SRC3-KO. Mice were exposed to CIH for 12 weeks. qRT-PCR was used to evaluate cardiac expression of the following genes: 11HSD1, 11HSD2, GR, MR, COX-2, OPN, NOX2, HIF-1-*α*, IL-1*β*, IL-6, iNOS, TNF-*α*, PC-1, and TGF-*β*. Enzymatic levels of SOD, CAT, MDA, NOS, and NO in the mouse hearts were determined using commercially available kits. Immunohistochemistry (IHC) was used to evaluate NF-*κ*B expression in cardiac tissues. A transmission electron microscope (TEM) was used to evaluate myocardial ultrastructure. TUNEL staining was used to assess myocardial cell apoptosis. CIH induced cardiac damage, which was ameliorated in the SRC-3 KO mice. CIH significantly increased the heart-to-body weight ratio, expression of all aforementioned genes except 11HSD1, GR, and MR, and increased the levels of MDA, NOS, NO, and NF-*κ*B, which were attenuated in the SRC-3 KO mice. The CIH group had the lowest SOD and CAT levels, which were partially recovered in the CIH+SRC3-KO group. 11HSD2 gene expression was elevated in both the CIH and CIH+SRC3-KO groups compared to the Ctrl group. The CIH group had severe myocardial cell apoptosis and mitochondrial dysfunction, which were alleviated in the CIH+SRC3-KO group. CIH causes cardiac damage through inducing oxidative stress and inflammation. Knockout of SRC-3 ameliorates CIH-induced cardiac damage through antagonizing CIH-triggered molecular changes in cardiac tissue.

## 1. Introduction

Sleep apnea syndrome (SAS) is prevalent in the general population and associated with cardiovascular diseases (CVDs) [1]. Chronic intermittent hypoxia (CIH) is a distinct pathophysiological feature of SAS and contributes to the development of SAS-associated CVDs [2]. CIH induces myocardial damage mainly through increasing oxidative stress and inflammation [3, 4], ultimately leading to CVD [5]. It should be noted that there is a positive feedback loop between oxidative stress and inflammation. Reactive oxygen species (ROS) induced by CIH activate hypoxia inducible fator-1*α* (HIF-1*α*), which in turn promotes sustained oxidative stress, further exacerbating myocardial inflammation [6] and consequently resulting in myocardial damage, atherosclerosis, and hypertension. CIH was also shown to increase vascular endothelial growth factor (VEGF) and endothelin-1 (ET-1) expression through HIF-1*α*, a pathophysiological process that may play an important role in the pathogenesis of SAS and cardiovascular damage [7]. Given that ROS, HIF-1*α*, and nuclear factor-*κ*B (NF-*κ*B) play a pivotal role in CIH-induced myocardial injury [6–8], it is critical to attenuate oxidative stress and inflammation to protect against CIH-induced myocardial injury.

Steroid receptor coactivator-3 (SRC-3) is a member of the p160 steroid receptor coactivator family and a coactivator of nuclear receptors (NR) and some transcription factors. The mineralocorticoid receptor (MR), as a member of the NR superfamily, binds to aldosterone and triggers oxidative stress and inflammation, eventually resulting in myocardial remodeling, fibrosis, and heart failure [9]. In recent years, SRC-3 has also been found to be involved in a variety of signal transduction pathways. For instance, SRC-3 overexpression promotes glycolysis in bladder cancer cells through activating HIF-1*α* [10]. SRC-3 has also been shown to promote inflammation by activating NF-*κ*B signaling pathways and promoting CXCL2 (CXC chemokine ligands 2) expression, thereby recruiting neutrophils [11]. SRC-3 deletion reduces NF-*κ*B nuclear translocation by inhibiting downregulation of I*κ*B-*α* (inhibitor of NF-*κ*B) levels in the early stages of inflammation, resulting in decreased expression of inflammatory factors including tumor necrosis factor-*α* (TNF-*α*), interleukin- (IL-) 2, IL-6, IL-8, and inducible nitric oxide synthase (iNOS) [12]. SRC-3 can also be recruited to the promoters of HIF-1*α* target genes. Given that HIF-1*α* is an important transcription factor involved in oxidative stress and that many of its downstream targets are closely related to CVDs, we postulate that SRC-3 contributes to the pathogenesis of CVDs through activating HIF-1*α* and NF-*κ*B, as well as promoting oxidative stress and inflammation.

In the present study, we investigated the mechanisms underlying CIH-induced myocardial injury and the involvement of SRC-3 in this pathology. We also explored the molecular basis related to SRC-3 function. We hypothesized that knockout of SRC-3 may protect the heart from CIH-induced damage by suppressing oxidative stress and inflammation.

## 2. Materials and Methods

### 2.1. Reagents

The following reagents were purchased from Roche: FastStart Universal SYBR Green Master ROX (2×) kit (Roche Cat# 4913914001), Tripure isolation reagent (Cat# 11667165001), FastStart Universal SYBR Master (Cat# 04913850001), and In Situ Cell Death Detection Kit (Cat# 11684817910). The ReverTra Ace quantitative real-time polymerase chain reaction (qPCR RT) kit was obtained from TOYOBO (Cat# FSQ-101). The Animal Total RNA Rapid Extraction Kit was from JieRui (Cat# GK3016). The RevertAid First Strand cDNA Synthesis Kit (K1622) was from Thermo Scientific (Cat# 1622). The following measurement kits were purchased from Nanjing Jiancheng Bioengineering Institute: superoxide dismutase (SOD) (Cat# A001-1), catalase (CAT) (Cat# A007-1), malondialdehyde (MDA) (Cat# A003-1), nitric oxide synthase (NOS) (Cat# A014-2), and NO (Cat# A012). Rabbit-anti-mouse NF-*κ*B antibody was from Abcam (Cat# Ab32536). The immunohistochemistry (IHC) and enhanced DAB chromogenic kits were obtained from Mai Xin.

### 2.2. SRC-3 Knockout (KO) Mice and Generation of the CIH Mouse Model

The animal protocol was approved by the Laboratory Animal Ethics Committee of the First Affiliated Hospital of Xiamen University. All methods were performed in accordance with ethical guidelines and regulations. This study was carried out in compliance with the ARRIVE guidelines. SPF grade SRC-3 KO mice on a Balb/c genetic background were generated at Baylor College of Medicine in the United States and donated by professor Chundong Yu, Life Sciences College, Xiamen University. SRC-3 KO mice were genotyped using PCR with the following oligos as previously described [13]: P76 5′GATGAGTGGACTAGGCGAAAGCT3 ′, P77 5′GCTGAGATTTGCAGAGATGAGTCC3 ′, and P78 5′GGCGATTAAGTTGGGTAACGCCAG3 ′.

A total of 24 SPF grade male mice, including 16 Balb/c wild type (WT) and 8 SRC3-KO (age, 8 to 10 weeks; body weight, 18~25 g) were divided into three groups: control (Ctrl), CIH, and CIH+SRC3-KO.

The CIH model was generated using an intermittent hypoxia system as described in our previous work [5, 7]. Mice in the CIH and CIH+SRC3-KO groups were placed in the CIH system. The Ctrl group underwent the same procedure as the other groups, except that these mice received only air.

In the CIH and CIH+SRC3-KO groups, CIH lasted for 8 h/day from 9:00-17:00 h for a duration of 12 weeks. All mice were anesthetized by intraperitoneal administration of 3% phenobarbital (30 mg/kg), perfused through the left ventricle with cold 100 mM phosphate buffer (pH 7.4). A small part of the apex of the heart was cut into small pieces within 1 mm cubes for transmission electron microscope (TEM) observation. A part of the heart was stored at −80°C for subsequent Western blot and qRT-PCR analyses. The remaining part of the heart was fixed in 10% neutral buffered formalin, stored in 70% ethanol, embedded in paraffin, and sectioned for subsequent histochemical assays.

### 2.3. Western Blot Analysis

Western blot analysis was performed on protein lysates purified from mouse hearts. Briefly, hearts were homogenized and lysates were purified in radioimmunoprecipitation assay (RIPA) buffer containing 1 mM phenylmethane sulfonylfluoride and a proteinase inhibitor cocktail (Roche). Protein concentrations were determined using a BCA protein quantification kit (Thermo). Equal amounts of total protein from each heart were separated using SDS-PAGE and transferred onto PVDF membranes (Millipore). The membranes were then immersed in 5% nonfat milk for 1 h and probed overnight with diluted primary antibodies against SRC-3 (1 : 1,000; Cell signaling) at 4°C. The membranes were then incubated with the appropriate HRP-conjugated antibody at room temperature for 2 h. Protein bands were visualized using an ECL reagent (Bio-Rad). GAPDH (Santa Cruz) was used as an internal control. The intensity of the specific band was quantified using ImageJ software and normalized to GAPDH.

### 2.4. qRT-PCR

qRT-PCR was performed to detect cardiac expression of the following genes: 11 beta-hydroxysteroid dehydrogenase type 1 and type 2 (11HSD1 and 11HSD2), glucocorticoid receptor (GR), MR, cyclooxygenase-2 (COX-2), osteopontin (OPN), NAPDH oxidase 2 (NOX2), HIF-1*α*, IL-1*β*, IL-6, iNOS, TNF-*α*, proprotein convertase 1 (PC-1), and transforming growth factor-*β* (TGF-*β*). Briefly, total RNAs were purified from mouse hearts of each group (*n* = 8 per group) using the Animal Total RNA Rapid Extraction Kit, and cDNA Synthesis was performed using the RevertAid First Strand cDNA Synthesis Kit. PCR was performed in a 20 *μ*L mixture consisting of 10 *μ*L of FastStart Universal SYBR Master, 2 *μ*L of Primer mix, and 8 *μ*L of template. GAPDH was used for normalization. Premier 5.0 software was used to design primers which were synthesized by Takara Bio Inc. (Takara, Japan). The primer sequences are listed in Table 1.

### 2.5. Measurement of Myocardial Enzymes

Total protein concentration in mouse myocardial homogenates was determined using the BCA protein quantification kit. SOD activity was measured using the xanthine oxidase method (hydroxylamine method). Malondialdehyde (MDA) was analyzed using the thiobarbituric acid method. NOS was measured using a colorimetry method. NO was detected using the nitrate reductase method. Catalase (CAT) was detected using the visible light method. The above methods were carried out according to the manufacturers' instructions.

### 2.6. IHC

IHC (The PV9001/DAB two-stage method) was used to determine NF-*κ*B cardiac expression. NF-*κ*B-positive cells were detected by pale yellow or brown staining. The average optical density values were calculated using IPP6.0 software.

### 2.7. TEM

Heart samples were fixed with 2% glutaraldehyde in 0.1 M phosphate-buffered saline (PBS) at 4°C overnight, followed by another fix with 0.5% potassium ferricyanide and 2% osmium tetroxide in 25 mM cacodylate buffer at 22°C. Samples were then dehydrated, infiltrated, and embedded in Spurr's resin. Sections were prepared at a thickness of 70 nm, poststained with lead citrate and uranyl acetate, and viewed under a JEM-2100HC TEM with a charge-coupled device camera (Japan Electronics Co., Ltd.). Images were captured at magnifications of 10,000~40,000.

### 2.8. TUNEL Assay

The In Situ Cell Death Detection Kit (Roche, POD, Cat# 11684817910) was used for TUNEL staining according to the instructions. Ten fields (×400) were randomly selected and scored, and the percentage of TUNEL-positive cells was determined by dividing the number of positively stained nuclei by the number of total nuclei in the field.

### 2.9. Statistical Analysis

Data are presented as the means ± standard deviation (SD). Data from multiple groups were compared with ANOVA followed by the Bonferroni post hoc test. A *p* value less than 0.05 was considered significant. Statistical analyses were performed using SPSS 20.0 and GraphPad Prism software.

## 3. Results

### 3.1. SRC-3 KO Ameliorated CIH-Induced Cardiac Hypertrophy

**Figure 1:**
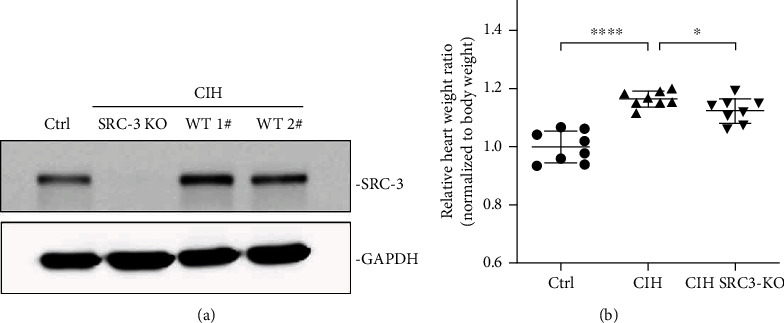
SRC-3 KO ameliorated CIH-induced cardiac hypertrophy. (a). Western blot analysis was used to evaluate the expression of SRC-3 in the mouse hearts from the three groups. GAPDH served as an internal control. (b) Heart weight/body weight ratios of mice were calculated. *N* = 8 per group. ^∗∗∗∗^*p* < 0.0001, ^∗^*p* < 0.05. Data are expressed as the means ± SD.

To examine the role of SRC-3 in CIH-induced cardiac hypertrophy, we first determined if CIH altered the expression of SRC-3 in mouse hearts. As shown in Figure 1(a), Western blot analysis revealed that CIH mice exhibited significantly higher cardiac SRC-3 expression compared to Ctrl mice. As expected, CIH induced cardiac hypertrophy as evaluated by the heart weight/body weight ratio (Figure 1(b), ^∗∗∗∗^*p* < 0.0001 vs. Ctrl), which was significantly attenuated in the SRC-3 KO mice (Figure 1(b), ^∗^*p* < 0.05*vs*. CIH). These findings indicate that CIH increases myocardial SRC-3 expression and that SRC-3 ablation protects the heart against CIH-induced cardiac hypertrophy.

### 3.2. SRC-3 KO Ameliorated CIH-Induced Pathological Changes in Cardiac Tissues

**Figure 2:**
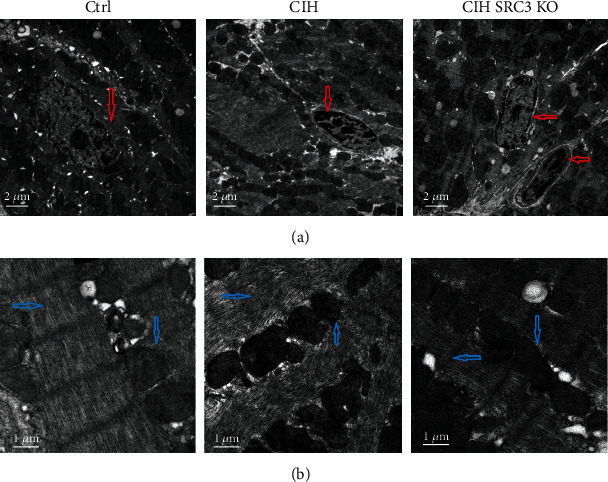
SRC-3 KO ameliorated CIH-induced myocardial pathological changes. (a) Pathological changes in myocardial cell nuclei in mice from the three groups were assessed using TEM. Red arrows point to the myocardial cell apoptosis in the CIH and CIH+SRC3-KO groups, while normal in the Ctrl group. Magnification: 10,000x, scale bar, 2 *μ*m. (b) Pathological changes in myocardial cell mitochondria and myonemes in mice from the three groups were assessed using TEM. Blue arrows point to the mitochondria structure, mitochondrial matrix, and coarse and fine filaments in three groups. Magnification: 40,000x; scale bar, 1 *μ*m.

We evaluated the pathological changes in the apical myocardium of mice using TEM. As expected, Ctrl mice had uniform chromatin in the myocardial nuclei and intact nuclear membranes (Figure 2(a)). However, the CIH group had pyknotic nuclei, disrupted nucleoli, and heterochromatin clustered in the perinuclear region. Analysis showed myocardial cell apoptosis in all CIH mouse hearts examined (*n* = 8). In the CIH+SRC3-KO group, the chromatin of the myocardial nuclei was mostly uniform and the nuclear membranes were intact, but some cardiomyocyte apoptosis was indicated by nuclear consolidation and nucleolus dissolution and disappearance. In addition, heterochromatin clusters around the nucleus were detected in 2 of the 8 CIH+SRC3-KO hearts (Figure 2(a)). These data suggest that SRC-3-KO alleviates the cardiac pathological changes induced by CIH.

Myocardial mitochondria and filaments were also evaluated using TEM. In the Ctrl mouse hearts, the mitochondria had a normal structure with intact membranes. The mitochondrial cristae were arranged in parallel and abundant in number. The electron density of the mitochondrial matrix was normal, and the coarse and fine filaments were well arranged, with clearly visible bands and Z lines (Figure 2(b)). However, in the CIH mouse hearts, the following mitochondrial effects were observed: the number of mitochondria decreased; sizes were abnormal and variable; shapes were malformed; some mitochondrial membranes were incomplete; cristae were reduced in number and were broken, dissolved, destroyed, flocculent, with widening gaps, and disordered; and the electron density of the mitochondrial matrix was increased. The coarse and fine filaments were loosely arranged and disordered, focally dissolved, the light and dark bands blurred, and the Z line disappeared, all of which were observed in the CIH hearts (*n* = 8) (Figure 2(b)). The ultrastructural changes in the mitochondria and myoneme in the CIH+SRC3-KO mouse hearts were similar to the Ctrl mouse hearts, in which the Z lines were still visible and there were few necrotic foci observed in the mitochondria.

### 3.3. SRC-3 KO Attenuated CIH-Induced Changes in Levels of Oxidative Stress-Related Enzymes

**Figure 3:**
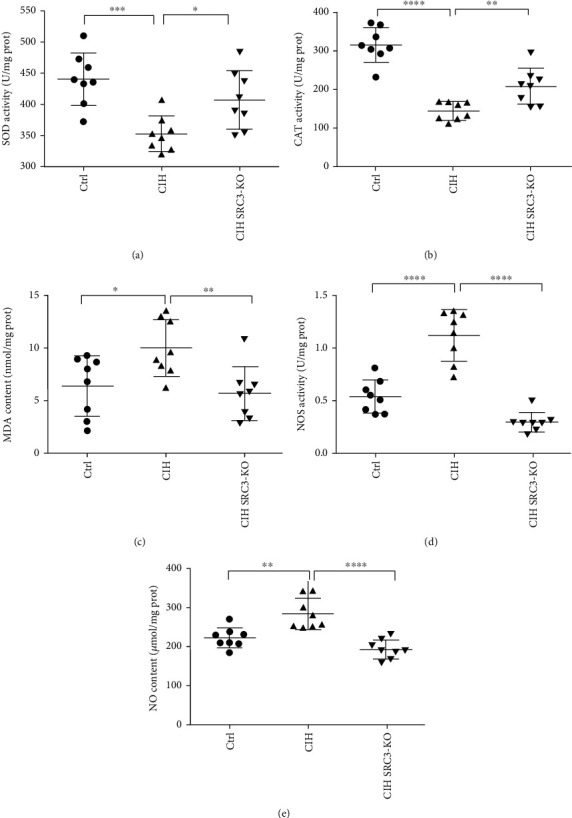
SRC-3 KO attenuated CIH-induced changes in level of oxidative stress-related enzymes in cardiac tissues. Assays were performed to measure the levels of the following enzymes in the mouse hearts from the three groups: SOD (^∗∗∗^*p* < 0.001, ^∗^*p* < 0.05), CAT (^∗∗∗∗^*p* < 0.0001, ^∗∗^*p* < 0.01), MDA (^∗^*p* < 0.05, ^∗∗^*p* < 0.01), and NOS (^∗∗∗∗^*p* < 0.0001). Myocardial NO levels were also measured (^∗∗^*p* < 0.01, ^∗∗∗∗^*p* < 0.0001). Data are expressed as the means ± SD. *N* = 8 per group.

We next examined whether CIH altered the levels of oxidative stress-related enzymes and the role of SRC-3 KO in mediating these changes. As shown in Figure 3, CIH significantly reduced the activity of SOD and CAT but increased the levels of MDA and iNOS. CIH also increased the levels of NO compared with the Ctrl mice. All of these changes were attenuated in the SRC-3 KO mice.

### 3.4. SRC-3 KO Attenuated CIH-Induced Transcription of Inflammatory and Fibrotic Genes

**Figure 4:**
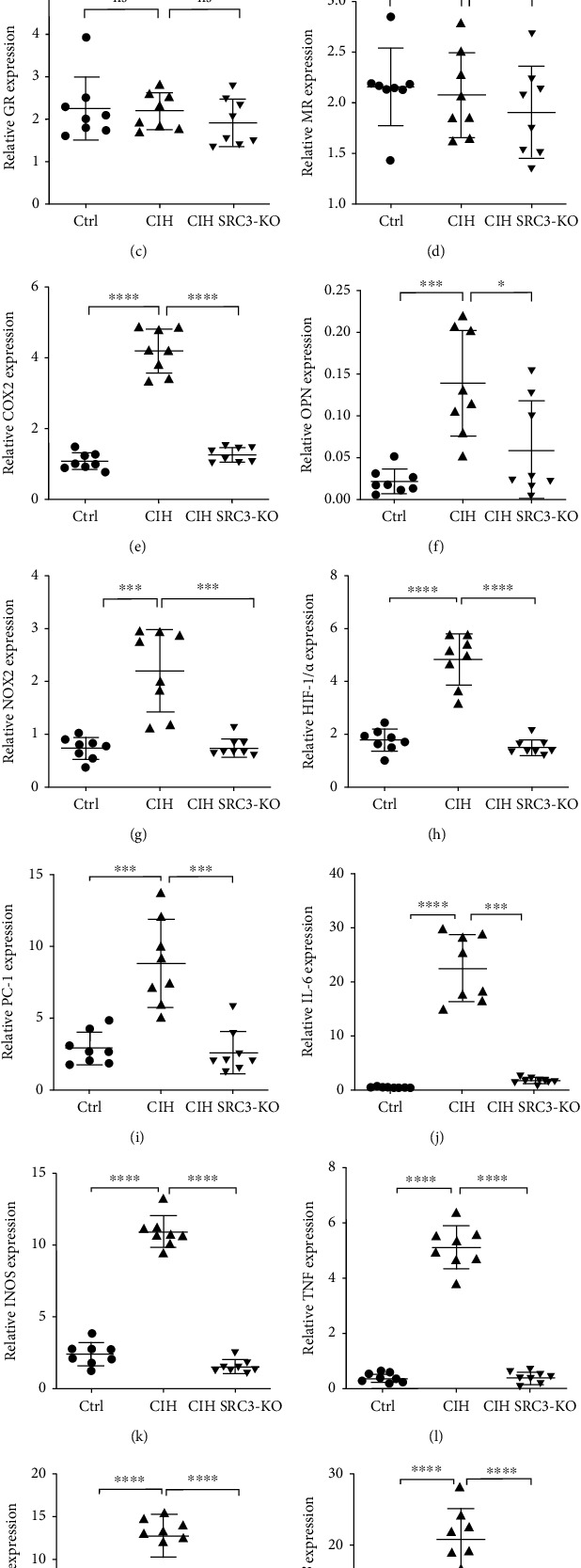
SRC-3 KO attenuated CIH-induced changes in transcription of inflammatory and fibrotic genes in cardiac tissues. qRT-PCR was performed to evaluate the transcription of the following inflammatory and fibrotic genes in the mouse hearts from the three groups: 11HSD1, 11HSD2, GR, MR, COX-2, OPN, NOX2, HIF-1*α*, IL-1*β*, IL-6, iNOS, TNF, PC-1, and TGF-*β*. GAPDH served as an internal control. ns: no significant difference. ^∗^*p* < 0.05, ^∗∗∗^*p* < 0.001, ^∗∗∗∗^*p* < 00001. Data are expressed as the means ± SD. *N* = 8 per group.

We next examined whether SRC-3 KO affected transcription of steroid hormones, inflammatory markers, and fibrotic genes induced by CIH using qRT-PCR. We measured expression of the following genes: 11HSD1, GR, MR, 11HSD2, COX-2, OPN, NOX2, HIF-1*α*, IL-1*β*, iNOS, TNF, PC-1, and TGF-*β*. As shown in Figure 4, CIH did not induce any significant changes in the expression of 11HSD1, GR, and MR, but it substantially increased the expression of all of the other genes. With the exception of 11HSD2, CIH-induced increases in gene transcription were significantly attenuated in the SRC-3 KO mice. These findings suggest that CIH-induced changes in the transcription of cardiac inflammatory and fibrotic genes are mediated in part through SRC-3.

### 3.5. SRC-3 KO Attenuated CIH-Increased NF-*κ*B Expression

**Figure 5:**
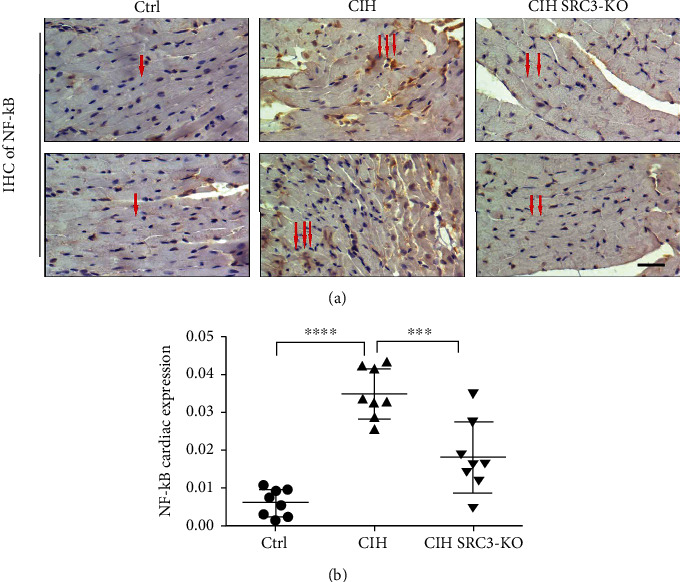
SRC-3 KO attenuated CIH-induced increases in NF-*κ*B cardiac expression. (a) IHC was performed to evaluate myocardial expression of NF-*κ*B in mice from the three groups. Yellow or brown staining indicates positive NF-*κ*B expression. Red arrows point to the NF-*κ*B staining in myocardial cells of mice. (b) The statistical analysis of (a). ^∗∗∗^*p* < 0.001, ^∗∗∗∗^*p* < 00001. Magnification: 400x; scale bar, 50 *μ*m.

We next evaluated the effect of CIH on the expression of NF-*κ*B, a critical transcriptional regulator of proinflammatory genes [14], in the mouse hearts using IHC. The CIH mice had significantly higher NF-*κ*B expression than the Ctrl mice, which was attenuated in the SRC-3 KO mice (Figures 5(a) and 5(b)).

### 3.6. SRC-3 KO Reduced CIH-Induced Myocardial Apoptosis

**Figure 6:**
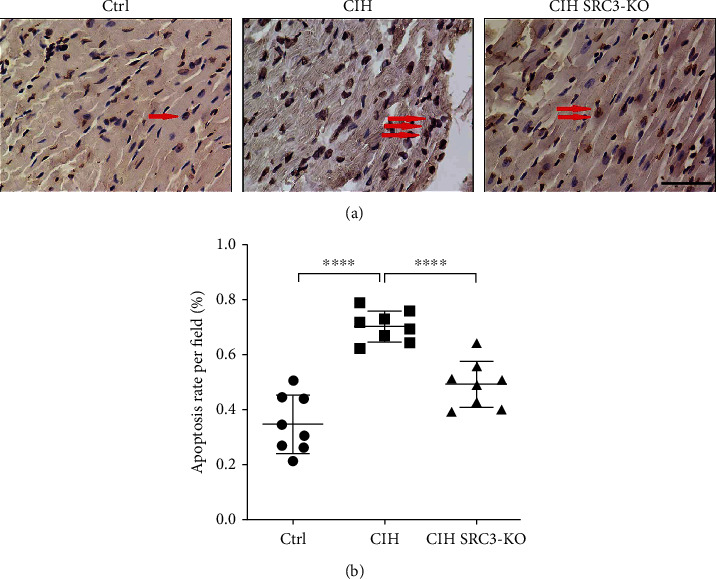
SRC-3 KO reduced CIH-induced myocardial apoptosis. (a) TUNEL staining was performed to assess myocardial cell apoptosis in mice from the three groups. Red arrows point to the myocardial cell apoptosis in mice. Data are expressed as the means ± SD. The CIH group exhibited the highest apoptosis rate compared to the other two experimental groups. (b) Quantification of (a). ^∗∗∗∗^*p* < 0.0001. Magnification: 400x; scale bar, 100 *μ*m. *N* = 8 per group.

We assessed myocardial cell apoptosis using the TUNEL assay. As shown in Figures 6(a) and 6(b), the CIH group had the highest apoptotic rate among the three groups (^∗∗∗∗^*p* < 0.0001), while the SRC-3 KO group had significantly reduced cardiomyocyte apoptosis (^∗∗∗∗^*p* < 0.0001).

## 4. Discussion

SAS is closely linked to the development of CVDs, and CIH plays an important role in this process. We used an SRC-3 loss of function model to investigate the role of SRC-3 in CIH-induced cardiac injury in mice. In our previous study, we have created a CIH system that mimics severe SAS [7, 15] demonstrating that no carbon dioxide accumulated.

The major findings from this study include the following: (1) SRC-3 expression was increased in mouse hearts subjected to CIH, (2) SRC-3 KO ameliorated CIH-induced pathological changes, including cardiac hypertrophy, and (3) mechanistically, SRC-3 KO attenuated CIH-induced apoptosis and changes in the activity of a number of oxidative stress-related enzymes and inflammatory genes in the mouse hearts.

It is well known that steroid hormones, including GR and MR, not only regulate electrolyte homeostasis but also play an important role in cardiovascular pathologies, such as myocardial remodeling [16, 17]. Interestingly, we found that CIH did not significantly affect the expression of GR and MR in the mouse hearts, nor did we observe any significant changes in 11HSD1 expression. However, CIH did affect 11HSD2 expression. 11HSD1 converts 11-oxo derivatives back to active glucocorticoids [18], which increases the local concentration of glucocorticoids, while 11HSD2 converts corticosterone and cortisol to their biologically inactive 11-oxo derivatives that have low affinity for MR [19]. MR mainly binds to aldosterone to induce its activation. Upregulation of 11HSD2 expression may contribute to mineralocorticoid-induced myocardial hypertrophy and remodeling [20], consistent with our finding.

Here, we found that transcription of the proinflammatory markers COX-2 and OPN was increased in the CIH-treated mouse hearts, which correlated with increased expression of the inflammatory and apoptosis marker NF-*κ*B in the hearts of mice subjected to CIH. It has been well documented that apoptosis contributes to the pathogenesis of a variety of CVDs, including cardiac hypertrophy [21]. Therefore, we speculate that increased apoptosis played a role in CIH-induced cardiac hypertrophy in our study.

Although CIH significantly increased 11HSD2 expression, SRC-3 KO did not affect 11HSD2 expression in the CIH mouse hearts. Therefore, whether 11HSD2 plays a role in SRC-3 KO-attenuated CIH-induced cardiac hypertrophy remains to be elucidated. SRC-3 KO alleviated the severity of cardiac hypertrophy and the proinflammatory markers COX-2 and OPN partially through attenuating myocardial cell apoptosis. This result is probably due to the reduced interaction of MR and SRC-3. As mentioned above, activation of MR by aldosterone results in upregulation of COX-2 and OPN [22, 23]. Thus, blocking the adverse effects of MR binding to aldosterone in the myocardium due to elevated levels of 11HSD2 could alleviate myocardial inflammation, hypertrophy, and remodeling caused by CIH.

CIH has been reported to cause oxidative stress and increase ROS levels [24, 25]. CIH can stimulate myocardial superoxide production via NADPH oxidase [26], a major source of ROS, to deteriorate left ventricular remodeling. Indeed, we observed that expression of NOX2, an NADPH oxidase and a biochemical marker of oxidative stress, significantly increased in the CIH mouse hearts. In contrast, CIH reduced the activity of SOD and CAT but increased the level of MDA in the mouse hearts. Therefore, we speculate that CIH-induced oxidative stress may be another mechanism leading to increased myocardial apoptosis and pathological changes. Interestingly, SRC-3 KO attenuated the CIH-induced changes in enzyme activity, which may represent another mechanism underlying SRC-3-mediated protection against CIH-induced cardiac hypertrophy. MR activation has been shown to mediate ROS production in many pathological conditions [27]. Spironolactone was demonstrated to inhibit aldosterone-mediated NADPH oxidase activation, suggesting that MR might be upstream of NADPH oxidase. SRC-3 is a coactivator of MR; thus, SRC-3 KO may alleviate myocardial oxidative stress damage [28].

Our results showed that CIH increased the expression of several inflammatory markers, including HIF-1*α*, IL-1*β*, IL-6, TNF, TGF-*β*, iNOS, PC-1, NO, and NF-*κ*B, all of which were attenuated by SRC-3 KO. Hypoxia has been widely reported as the most powerful inducer of the HIF-1*α* transcriptional activity [29], which in turn activates the transcription of over 200 genes [30], many of which are closely related to the CIH-induced inflammatory response (IL-1*β*, IL-6 [31], NOS [32], TNF-*α* [33], PC-1 [34], and NF-*κ*B [35]). IL-1*β* is a potent proinflammatory cytokine that can also induce expression of downstream proinflammatory molecules, such as COX-2 and NO [36]. iNOS produces NO, which is a messenger molecule with diverse functions in inflammation and synthesis of proinflammatory mediators (i.e., IL-6 and IL-8). TNF-*α* is a proinflammatory cytokine that regulates a wide spectrum of biological processes, including cell proliferation, differentiation, apoptosis, lipid metabolism, and coagulation. PC-1 is a fibrotic marker that is secreted as abundant collagen I into the extracellular matrix after procollagen is modified by prolylhydroxilase [34]. TGF-*β* is another fibrotic marker that regulates cell proliferation, differentiation, and growth and can modulate expression and activation of other growth factors (i.e., TNF-*α*). In addition, there is a positive feedback loop between TGF-*β* and ROS [37, 38]; TGF-*β* induces and stabilizes HIF-1*α* expression, activates fibroblasts, and promotes collagen deposition [39]. On the other hand, there is a complex relationship between ROS, HIF-1*α*, and NF-*κ*B and other inflammatory factors. HIF-1*α* directly regulates the expression of NF-*κ*B, which activates many inflammatory factors (i.e., TNF-*α*, TGF-*β*, IL-6, IL-8, COX-2, and iNOS) [40]. ROS can stimulate the production of inflammatory factors, such as NF-*κ*B, activator protein-1, and HIF-1*α*. Moreover, NF-*κ*B is closely related to apoptosis. Taken together, our data indicate that increased inflammation plays an important role in CIH-induced cardiac injury and that SRC-3 can partially reduce inflammation.

TEM revealed that CIH mouse hearts had severe myocardial cell apoptosis consistent with TUNEL assay results and abnormal ultrastructural changes in the mitochondria and cristae. These effects were ameliorated in the SRC-3 KO mice. Combined with the aforementioned findings, we propose that CIH induces cardiac injury through multiple mechanisms, including increasing oxidative stress and inflammation. These pathophysiological changes lead to elevated apoptosis and changes in myocardial ultrastructure. It should be noted that these molecular mechanisms and pathophysiological changes should not be viewed as individual independent processes; instead, they should be viewed as a part of a larger interconnected process.

SRC-3 belongs to the p160 steroid receptor coactivator family and is particularly sensitive to changes in intracellular signaling [41]. SRC-3 is ubiquitously expressed in various organs, including the heart [42, 43]. In the present study, we observed that SRC-3 KO mice exhibited reduced apoptosis, ultrastructural changes, and cardiac hypertrophy compared with CIH mice, which was accompanied by reduced expression of the inflammatory genes and oxidative enzymes. Therefore, SRC-3 mediates CIH-induced cardiac pathological changes through multiple mechanisms.

**Figure 7:**
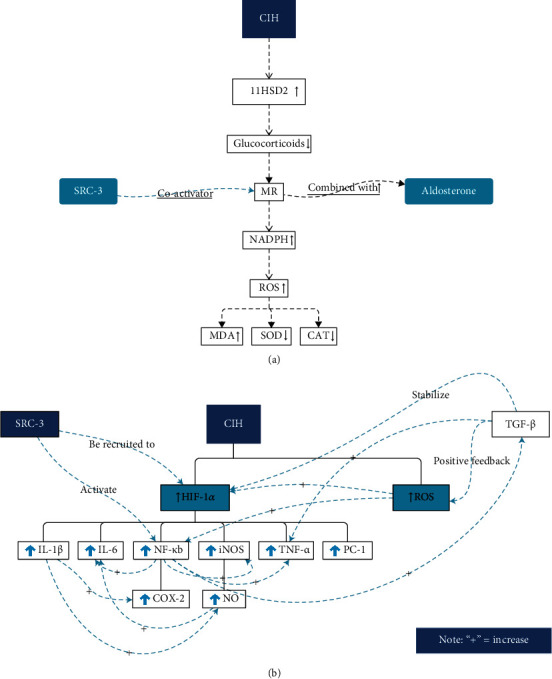
Hypothesis and working model for the SRC-3 function in CIH-induced myocardial injury. (a) CIH may induce myocardial oxidative stress injury via SRC-3 coactivating MR. (b) CIH may induce transcription of inflammatory and fibrotic genes and oxidative stress in the heart via activated SRC-3.

Previous studies have proved that overexpression of SRC-3 promotes glycolysis through activating HIF-1*α* and aggravates inflammation by activating NF-*κ*B signaling pathways [10, 11], while SRC-3 deletion reduces NF-*κ*B nuclear translocation, inflammatory, and iNOS [12]. Besides, SRC-3 can also be recruited to the promoters of HIF-1*α* target genes [44], and many of its downstream target genes (i.e., IL-1*β*, IL-6, iNOS, TNF, PC-1, and NF-*κ*B) as demonstrated in this study. Furthermore, HIF-1*α* is an important transcription factor involved in oxidative stress and that many of its downstream targets are closely related to CVDs [6]. It is easy to come up with SRC-3 may regulate the downstream NF-*κ*B signaling pathways [45] (Figures 7(a) and 7(b)), inflammatory, and iNOS by recruitment to the promoters of HIF-1*α*. We postulate that SRC-3 contributes to the pathogenesis of CVDs through activating HIF-1*α* and NF-*κ*B, as well as promoting oxidative stress and inflammation.

In the present study, we used the heart weight/body weight ratio to evaluate the severity of cardiac hypertrophy. Although this index has been widely used in the field [46], it would be optimal to combine this index with cardiac functional parameters obtained by echocardiography to better evaluate cardiac hypertrophy.

Although we found that CIH increased the levels of myocardial fibrosis markers, we did not observe significant changes in myocardial fibrosis using electron microscopy. This might be because myocardial fibrosis is a late change in myocardial injury. This disparity should be clarified in a prolonged chronic intermittent hypoxia model, such as more than 12 weeks.

## 5. Conclusions

In summary, we found that SRC-3 KO ameliorates CIH-induced cardiac hypertrophy in mice, which is associated with attenuated myocardial oxidative stress and inflammation. Hence, SRC-3 is a potentially new target for the treatment of CVDs caused by SAS.

## Figures and Tables

**Table 1:** qRT-PCR primer sequences.

Primer name	Primer sequence (5′-3′)
MusTNF-F	CACAGAAAGCATGATCCGCG
MusTNF-R	ACTGATGAGAGGGAGGCCAT
MusIL-1*β*-F	GCAGTGGTTCGAGGCCTAAT
MusIL-1*β*-R	GCTGCGAGATTTGAAGCTGG
MusIL-6-F	AGCCAGAGTCCTTCAGAGAGA
MusIL-6-R	GGATGGTCTTGGTCCTTAGCC
MusiNOS-F	AGAATCCCTGGACAAGCTGC
MusiNOS-R	TTGTCTCTGGGTCCTCTGGT
MusCOX-2-F	TCCCCATTAGCAGCCAGTTG
MusCOX-2-R	TGCTCATACATTCCCCACGG
MusNOX2-F	GGGAACTGGGCTGTGAATGA
MusNOX2-R	CTGGCAGCAGGATCAGCATA
MusHIF-1*α*-F	TCATCAGTTGCCACTTCCCC
MusHIF-1*α*-R	TGTAAACCATGTCGCCGTCA
MusPC-1-F	CCAGCCGCAAAGAGTCTACA
MusPC-1-R	GGGTTTCCACGTCTCACCAT
MusTGF-*β*-F	CACCTTTGCCGAGGGTTCC
MusTGF-*β*-R	GTTTCACCAGCTCCATGTCG
Mus11HSD1-F	GTGTCTCGCTGCCTTGAAC
Mus11HSD1-R	ACCTCCATGACTCTTCGCAC
Mus11HSD2-F	TGACCAAGGCAGAGGACATC
Mus11HSD2-R	ACTGGAGACAGTTCCACGTC
MusOPN-F	TCTCAGAAGCAGAATCTCCTTGC
MusOPN-R	ATGTGGTCATGGCTTTCATTGG
MusGR-F	GGGGCTATGAACTTCGCAGG
MusGR-R	CTTCATCGGAGCACACCAGG
MusMR-F	TGAGTTCCTTTCCGCCTGTC
MusMR-R	CTCATCTCCACACACCAAGCAG
MusGAPDH-F	AGGCCGGTGCTGAGTATGTC
MusGAPDH-R	TGCCTGCTTCACCACCTTCT

## Data Availability

The datasets used and/or analyzed during the current study are available from the corresponding author on reasonable request.

## Abbreviations

CIH: Chronic intermittent hypoxia
SAS: Sleep apnea syndrome
CVDs: Cardiovascular diseases
SRC-3: Steroid receptor coactivator -3
WT: Wild type
SRC3-KO: SRC-3 knockout
Ctrl: Air control
IHC: Immunohistochemistry
TEM: Transmission electron microscope
TUNEL: TdT-mediated dUTP nick end labeling
DAB: Diaminobenzidine
RIPA: Radioimmunoprecipitation assay
ROS: Reactive oxygen species
HIF-1*α*: Hypoxia-inducible fator-1*α*
NF-*κ*B: Nuclear factor-*κ*B
NR: Nuclear receptors
GR: Glucocorticoid receptor
MR: Mineralocorticoid receptor
TNF-*α*: Tumor necrosis factor-*α*
IL: Interleukin
iNOS: Inducible nitric oxide synthase
NOS: Nitric oxide synthase
SOD: Superoxide dismutase
CAT: Catalase
MDA: Malondialdehyde
11HSD1 and 11HSD2: 11 Beta-hydroxysteroid dehydrogenase type1 and type 2
COX-2: Cyclooxygenase-2
OPN: Osteopontin
NOX2: NAPDH oxidase 2
PC-1: Proprotein convertase 1
TGF-*β*: Transforming growth factor-*β*
CXCL2: CXC chemokine ligands 2
I*κ*B-*α*: Inhibitor of NF-*κ*B
ANOVA: Analysis of variance.
